# Trends in Hypertension Prevalence, Awareness, Treatment and Control in an Adult Type 2 Diabetes Spanish Population between 2003 and 2009

**DOI:** 10.1371/journal.pone.0086713

**Published:** 2014-01-27

**Authors:** Carmen de Burgos-Lunar, Rodrigo Jiménez-García, Miguel A. Salinero-Fort, Paloma Gómez-Campelo, Ángel Gil, Juan C. Abánades-Herranz, Juan Cárdenas-Valladolid, Isabel del Cura-González

**Affiliations:** 1 Clinical Epidemiology and Research Unit, Carlos III Hospital, Madrid, Spain; 2 Red de Investigación en Servicios de Salud en Enfermedades Crónicas (REDISSEC), Madrid, Spain; 3 Preventive Medicine and Public Health Department, Rey Juan Carlos University, Alcorcón, Spain; 4 Biomedical Research Foundation, Carlos III Hospital, Madrid, Spain; 5 Research Unit, Dirección Técnica de Docencia e Investigación, Gerencia Atención Primaria, Madrid, Spain; CAEBi, Spain

## Abstract

In patients with type 2 diabetes, the prevalence of hypertension is higher than in non-diabetic subjects. Despite the high cardiovascular risk involving hypertension in these patients, its prevalence and control are not well known. The aims of this study were: to estimate the hypertension prevalence, awareness, treatment and control in Spanish adults with type 2 diabetes attended in Primary Care; and to analyse its time trend from 2003 to 2009. A serial cross-sectional study from 2003 to 2009 was performed in 21 Primary Care Centres in Madrid. The study population comprised all patients with diagnosed type 2 diabetes in their computerised medical history. Overall annual prevalence during the period 2003–2009 was calculated from and according to sex and age groups. Linear trend tests, regression lines and coefficients of determination were used. In 2003 89.78% (CI 87.92–91.64) of patients with type 2 diabetes suffered hypertension and 94.76% (CI: 92.85–96.67) in 2009. This percentage was greater for women and for patients over 65 years old. 30% of patients suffered previously undiagnosed hypertension in 2003 and 23.1% in 2009. 97% of diagnosed patients received pharmacological treatment and 28.79% reached the blood pressure objective in 2009. The average number of antihypertensive drugs taken was 2.72 in 2003 and 3.27 in 2009. Only 5.2% of patients with type 2 diabetes show blood pressure levels below 130/80 mmHg. Although significant improvements have been achieved in the diagnosis and control of hypertension in people with type 2 diabetes, these continue to remain far from optimum.

## Introduction

Arterial hypertension (HTN) is a cardiovascular risk factor which affects almost 40% of the adult population of the developed countries [1], [2]. In Spain, this prevalence is near 35% in adults, reaching 68% in those over 60 years old [3].

The prevalence of HTN in patients with type 2 diabetes is between 1.5 and 2.3 times greater than for non-diabetic subjects [4], [5]. The prognostic implications of the coexistence of diabetes and HTN are greater than those constituted by each independent condition. Between 70% and 80% of people with diabetes die as a result of cardiovascular complications, 75% of which can be attributed to HTN [6], [7].

Several clinical trials [8]–[10] have provided evidence that intensive treatment of HTN reduces mortality and prevents or delays the incidence of micro-vascular and macro-vascular complications in people with diabetes. The control of HTN in diabetic patients has achieved a reduction in cardiovascular and renal complications higher than in non-diabetic hypertensive population. Moreover, for patients with diabetes the benefits of a strict control of blood pressure (BP) are greater than the benefits of a of tight glycemic control [9].

There is no agreement in setting a BP diagnostic threshold for HTN in diabetic patients. Due to their increased cardiovascular risk, many scientific societies have set up a lower threshold (BP≥130/80 mmHg) than that set for patients without diabetes (BP≥140/90 mmHg) [11]–[14]. Nevertheless, the European Guide to the Management of HTN in its 2009 revision [15], that were still in effect in 2013 [16], raises the cut-off point which it fixed in 2007 at 130/80 mmHg to 140/85 mmHg; and the National Institute for Health and Clinical Excellence (NICE) maintains the threshold at the same values as for people without diabetes [17].

In the Spanish National Health System, the medical care responsibility for the prevention, diagnosis, treatment and control of diabetes and HTN devolves mainly upon general practitioners in Primary Care (PC), making this the most suitable field in which to obtain information in real conditions of clinical practice.

Care protocols for stable diabetic patients in PC recommend two visits to the general practitioners each year and three or four nursing visits [18].

All health centres in Madrid have computerized clinical records available since 2002 and the diagnoses of HTN and diabetes recorded in these have been validated [19]. This has allowed us to rely on an excellent source of information to investigate the prevalence of the different pathologies and for evaluating the subsequent monitoring of patients.

The objectives of this study are: 1) to estimate the HTN prevalence, awareness, treatment and control in Spanish adults with type 2 diabetes and for subsets by age and sex, 2) to analyse its temporal evolution during the period 2003 to 2009.

## Methods

### Design

Serial cross-sectional study from 2003 to 2009.

### Setting

The study was carried out in the 21 PC Centres of the health area 4, in the northeast urban zone of the Community of Madrid.

Over the study period the population which serves the Area 4 increased by 15.6% and the prevalence of people with type 2 diabetes rose from 5.12% to 8.66%.

### Population

All patients monitored in PC from 2003 to 2009 with a diagnosis of type 2 diabetes in their Computerized Clinical Records (CCR) were included if they met the following inclusion criteria: over 18 years of age and had visited their PC Health Centres at least twice a study-year. Patients were not included if they had not at least 2 measurements of BP recorded per study-year.

### Sources of Information

Information was obtained from individualised data contained in the CCR of the patients. We collected socio-demographic and clinical variables, care procedures, treatments and laboratory results.

### Variables

Diagnosed HTN: diagnostic record of HTN in medical history (code K86 or K87 of the International Classification of PC).

Undiagnosed HTN: the average of two or more determinations of systolic BP on at least 2 visits was ≥130 mmHg or the average of diastolic BP was ≥80 mmHg [11], [12], [14] without a diagnostic record.

To be able to tackle our investigation question, and in face of the lack of agreement between different scientific societies in establishing HTN diagnostic thresholds for diabetic patients, two HTN thresholds were established: undiagnosed HTN with BP values between 130/80 mmHg and 140/90; and undiagnosed HTN with BP values ≥140/90 mmHg.

In patients with diagnosed HTN a record of pharmacological treatment was compiled (number and class of drugs), and the HTN control considering values less than 130/80 mmHg and 140/90 as the objectives. Patient demographic, anthropometric, and clinical characteristics were also recorded.

Since the diagnostic criteria established by the European Guidelines for the Management of hypertension in November 2009 [15] were not in force at the time the study was conducted, they were not taken into account in the analysis. Nevertheless, the results with the cutoff set at this guide had been included as Supporting Information (Table S1).

### Statistical Analysis

A descriptive analysis of the main demographic and clinical characteristics of the study population was carried out. Annual prevalence were calculated during the period 2003–2009.

Linear trend tests were used and regression lines with their coefficients of determination (R^2^) were calculated for each category. All analyses were calculated with their confidence interval of 95% (CI) overall and stratified by sex and age groups. Statistical significance was set at *p*<0.05.

Statistical processing of the data was performed with SPSS 15.0® software (SPSS Inc., Chicago, IL).

### Ethical Aspects

The study has been developed in accordance with that established by current legislation and complies with the norms of good clinical practice.

Informed consent was not necessary as personal identifying information was kept separate from the research data, and patients will not be identifiables and guarantees complete confidentiality of the clinical information that is obtained in compliance with Law 15/1999, of 13 December, on the Protection of Personal Data and Law 41/2002, of 14 November, on the autonomy of the patient and the rights and obligations pertaining clinical information and documentation.

The study protocol was approved by the Ethics Committee of the Carlos III Hospital in Madrid.

## Results

Our study compiles available information on all the people with diabetes attended in the health area who met the inclusion criteria. The percentage of excluded patients that had not gone to their PC Health Centre at least twice a year nor had at least 2 measurements of BP was 10.3% in 2003, 4.7% in 2004, 5.6% in 2005, 12.4% in 2006, 8% in 2007, 6% in 2008 and 2.7% in 2009.

Since the population which serves the health area and the prevalence of type 2 diabetes increased during the study period, diabetic patients included rose from 10,517 in 2003 to 22,123 in 2009.

The baseline characteristics of subjects are shown in Table 1.

**Table 1:** Baseline characteristics of the study group between 2003 and 2009.

		2003	2004	2005	2006	2007	2008	2009
		(n = 10517)	(n = 13075)	(n = 15323)	(n = 17221)	(n = 19177)	(n = 20934)	(n = 22123)
Gender	(% Female)	53	52.6	52.2	51.8	51	50.4	50.3
Age (years)	Mean (SD)	67.7 (10.4)	68.1 (10.6)	68.7 (10.8)	69.4 (11)	69.8 (11.2)	70.3 (11.4)	71 (11.5)
Duration of diabetes (years)	Mean (SD)	4.8 (6.7)	4.9 (6.6)	5.1 (6.4)	5.5 (6.4)	5.9 (6.4)	6.4 (6.4)	7 (6.4)
BMI (Kg/m^2^)	Mean (SD)	29.9 (4.8)	30.1 (4.9)	30 (4.9)	30 (4.8)	30 (4.9)	30 (4.9)	29.9 (4.9)
BMI	(%)							
<25 kg/m^2^		12.9	12.7	12.8	12.9	13.1	13.3	14
25–30 kg/m^2^		41.8	41.8	41.9	41.9	41.5	42.4	42.7
≥30 kg/m^2^		45.3	45.4	45.3	45.2	45.4	44.3	43.4
Systolic blood pressure (mmHg)	Mean (SD)	134.9 (13.9)	134.3 (13.4)	133.8 (13.2)	133.3 (13.4)	134.1 (13.5)	133.2 (13.2)	132.6 (13)
Diastolic blood pressure (mmHg)	Mean (SD)	77.6 (7.9)	77 (8)	76.7 (7.8)	76.4 (8)	76.5 (7.9)	76 (7.9)	75.5 (7.8)
Hypertension	(%)	62.86	64.55	66.33	68.74	70.06	71.26	72.85
Smoking habit	(%)							
Never smokes		80.6	77.7	76.2	75.1	73.4	72.3	71.9
Ex-smoker		2.1	2	2.2	2.4	2.6	3.5	5
Smoker		17.3	20.3	21.6	22.5	24	24.2	23.1
HbA1c (%)	Mean (SD)	7.2 (1.4)	7.2 (1.3)	7.3 (1.3)	7.2 (1.2)	7.1 (1.2)	6.9 (1.2)	7 (1.1)
Type of diabetes treatment	(%)							
Only diet		6.2	7	8.1	8.8	9.4	11.5	10.2
Oral antidiabetic agents		40.7	37.5	35.4	34.2	31.6	32	32
Insulin		7	6.6	5.9	5.8	5.6	5.4	5.8
Oral antidiabetic agents +insulin		46.1	48.9	50.6	51.2	53.5	51.1	52
Triglycerides	Mean (SD)	149.6 (95.9)	143.2 (92.3)	138.6 (86.3)	141.6 (98.7)	143.1 (90.8)	140.2 (101.7)	142 (88.3)
Cholesterol	Mean (SD)	207.3 (35.7)	203.9 (35.4)	200.2 (35.4)	196.9 (35.7)	196 (36.1)	191.7 (35.9)	187.2 (35.7)
LDL cholesterol	Mean (SD)	129.7 (31.7)	126.6 (30.3)	124.1 (30.2)	121.3 (31.1)	119.3 (31.2)	114.8 (30.5)	109.8 (29.8)
HDL cholesterol	Mean (SD)	48.2 (12.9)	49.4 (12.2)	48.8 (11.9)	47.8 (11.9)	48.1 (12)	48.7 (12.3)	48.9 (12.4)
Albuminuria	Mean (SD)	18.7 (46.2)	20.5 (53.4)	19.3 (50.9)	17.8 (52.6)	14.6 (50)	26.7 (72.6)	30.2 (81.1)
Microalbuminuria	(%)	27.4	27.4	29.5	34.9	36.8	43.9	38.9
Glycemic control (HbA1c <7%)	(%)	50	49.3	47.8	51.1	55.2	61.6	59.6
Systolic blood pressure <140 mmHg	(%)	64.1	66.4	68	69.4	67	69.9	71.8
Systolic blood pressure <130 mmHg	(%)	31.7	33.8	35.6	37.1	34.9	37.6	39.5
Diastolic blood pressure <90 mmHg	(%)	93.2	93.8	94.4	94.2	94	94.9	95.2
Diastolic blood pressure <80 mmHg	(%)	56.7	60	62.7	63.8	63	66	67.9
Blood pressure control (<140/90 mmHg)	(%)	62.8	65.1	66.9	68.4	65.9	68.9	70.9
Blood pressure control (<130/80 mmHg)	(%)	25.3	27.8	29.5	31.5	29.5	32.4	34.4
Antihypertensive drugs	(%)	85.2	84.9	84.2	84.1	83.5	83	82.8

SD: standard deviation.

The prevalence of HTN in patients with type 2 diabetes in 2003 was 89.78% (CI: 87.92–91.64). This prevalence showed a significantly (p<0.001) annual increase of 0.64% during the study period, reaching 94.76% (CI: 92.85–96.67) in 2009. This percentage was higher for women and for patients over 65 years old.

If the diagnostic threshold for BP is established at 140/90 mmHg, the prevalence of HTN during the years 2003–2009 rose significantly (p<0.001) from 69.77% (CI: 68.14–71.41) to 79.87% (CI: 78.12–81.62).

The percentage of undiagnosed HTN declined significantly (p<0.001) by 22.9%, falling from 30% in 2003 to 23.1% in 2009. This reduction is attributable to the group of patients with BP between 130/80 mmHg and 140/90 mmHg, given that the proportion of people with BP≥140/90 mmHg remains practically constant.

The percentage of patients with diagnosed HTN who show BP<130/80 mmHg rose from 16.71% in 2003 to 28.73% in 2009. This control objective was achieved for 25.3% of all type 2 diabetes patients with arterial HTN (diagnosed or undiagnosed) in 2003 and for 34.4% in 2009. If the control objective is set at a BP of <140/90 mmHg, in 2003 this was reached by 53.03% of patients diagnosed with HTN and in 2009 by 65.77%.

Table 2 shows the global and stratified by sex and age groups annual prevalence of HTN, control and treatment and the trend analysis for the study period.

**Table 2:** Prevalence, awareness, treatment and control of hypertension among adults with diabetes by sex and age groups between 2003 and 2009.

PREVALENCE AND AWARENESS								
TOTAL	(n = 10517)	(n = 13075)	(n = 15323)	(n = 17221)	(n = 19177)	(n = 20934)	(n = 22123)	
Without hypertension	10.22 (9.59–10.85)	9.09 (8.5–9.68)	8.14 (7.59–8.7)	7.28 (6.75–7.8)	6.46 (5.96–6.95)	5.84 (5.37–6.34)	5.24 (4.79–5.69)	<0.001
Undiagnosed hypertension (<140/90 mmHg)	20.01 (19.13–20.88)	19.40 (18.54–20.27)	18.38 (17.54–19.22)	17.02 (16.21–17.83)	16.32 (15.52–17.11)	15.77 (14.99–16.56)	14.89 (14.13–15.65)	<0.001
Undiagnosed hypertension (≥140/90 mmHg)	6.91 (6.4–7.43)	6.95 (6.44–7.47)	7.15 (6.63–7.68)	6.97 (6.45–7.49)	7.17 (6.65–7.69)	7.13 (6.6–7.65)	7.02 (6.5–7.54)	0.605
Diagnosed hypertension	62.86 (61.31–64.41)	64.55 (62.98–66.13)	66.33 (64.73–67.92)	68.74 (67.11–70.36)	70.06 (68.42–71.7)	71.26 (69.61–72.9)	72.85 (71.17–74.52)	<0.001
**FEMALES**	**(n = 5576)**	**(n = 6883)**	**(n = 7993)**	**(n = 8929)**	**(n = 9782)**	**(n = 10544)**	**(n = 11126)**	
Without hypertension	7.44 (6.91–7.98)	6.92 (6.4–7.43)	6.11 (5.62–6.59)	5.23 (4.78–5.68)	4.63 (4.21–5.05)	4.36 (3.95–4.78)	3.83 (3.45–4.21)	<0.001
Undiagnosed hypertension (<140/90 mmHg)	16.70 (15.9–17.5)	15.40 (14.63–16.17)	14.80 (14.05–15.55)	13.67 (12.95–14.4)	13.08 (12.37–13.78)	12.25 (11.57–12.96)	11.57 (10.9–12.23)	<0.001
Undiagnosed hypertension (≥140/90 mmHg)	5.94 (5.46–6.41)	6.16 (5.67–6.65)	6.09 (5.61–6.58)	6.00 (5.52–6.48)	5.84 (5.36–6.31)	5.69 (5.22–6.16)	5.80 (5.33–6.27)	0.120
Diagnosed hypertension	69.92 (68.29–71.56)	71.52 (69.87–73.18)	73.00 (71.33–74.68)	75.09 (73.39–76.79)	76.46 (74.74–78.17)	77.69 (75.97–79.41)	78.81 (77.07–80.55)	<0.001
**MALES**	**(n = 4941)**	**(n = 6192)**	**(n = 7330)**	**(n = 8292)**	**(n = 9395)**	**(n = 10390)**	**(n = 10997)**	
Without hypertension	13.36 (12.64–14.07)	11.51 (10.85–12.18)	10.37 (9.74–11)	9.48 (8.88–10.08)	8.36 (7.79–8.92)	7.34 (6.81–7.91)	6.67 (6.16–7.17)	<0.001
Undiagnosed hypertension(<140/90 mmHg)	23.74 (22.79–24.7)	23.85 (22.9–24.81)	22.28 (21.35–23.2)	20.62 (19.73–21.51)	19.69 (18.82–20.56)	19.34(18.47–20.21)	18.25 (17.41–19.09)	<0.001
Undiagnosed hypertension (≥140/90 mmHg)	8.01 (7.46–8.57)	7.83 (7.28–8.38)	8.31 (7.74–8.87)	8.01 (7.45–8.56)	8.56 (5.26–6.41)	8.59 (8.01–9.16)	8.27 (7.7–8.83)	0.156
Diagnosed hypertension	54.89 (53.44–56.34)	56.80 (55.32–58.28)	59.05 (57.54–60.55)	61.89 (60.35–63.43)	63.40 (74.9–78.02)	64.74(63.16–66.3)	66.82 (65.22–68.42)	<0.001
**AGE <65 years**	**(n = 3663)**	**(n = 4474)**	**(n = 5024)**	**(n = 5361)**	**(n = 5950)**	**(n = 6257)**	**(n = 6257)**	
Without hypertension	15.37 (14.24–16.57)	13.5 (12.53–14.53)	12.66 (11.77–13.61)	11.3 (10.48–12.18)	10.84 (11.66–10.08)	9.89 (10.66–9.18)	9.05 (9.78–8.36)	<0.001
Undiagnosed hypertension (<140/90 mmHg)	24.22 (22.86–25.63)	24.79 (23.54–26.07)	24.32 (23.16–25.53)	22.85 (21.75–23.99)	22.82 (23.91–21.77)	22.98 (24.04–21.96)	22.18 (23.23–21.17)	<0.001
Undiagnosed hypertension (≥140/90 mmHg)	6.63 (5.87–7.49)	6.33 (5.65–7.08)	6.81 (6.14–7.54)	6.68 (6.04–7.38)	7.24 (7.93–6.61)	7.21 (7.88–6.59)	7.11 (7.78–6.5)	0.052
Diagnosed hypertension	53.78 (52.16–55.39)	55.39 (53.93–56.84)	56.21 (54.83–57.58)	59.17 (57.85–60.48)	59.09 (60.34–57.84)	59.92 (61.12–58.7)	61.66 (62.86–60.45)	<0.001
**AGE ≥65 years**	**(n = 6854)**	**(n = 8601)**	**(n = 10299)**	**(n = 11860)**	**(n = 13227)**	**(n = 14677)**	**(n = 15866)**	
Without hypertension	7.47 (6.87–8.12)	6.8 (6.29–7.35)	5.94 (5.5–6.42)	5.46 (5.06–5.88)	4.48 (4.14–4.85)	4.12 (3.81–4.45)	3.74 (3.45–4.04)	<0.001
Undiagnosed hypertension (<140/90 mmHg)	17.76 (16.87–18.68)	16.6 (15.83–17.4)	15.48 (14.79–16.19)	14.38 (13.76–15.03)	13.39 (12.82–13.98)	12.69 (12.16–13.24)	12.01 (11.52–12.53)	<0.001
Undiagnosed hypertension (≥140/90 mmHg)	7.06 (6.48–7.69)	7.28 (6.75–7.85)	7.32 (6.83–7.84)	7.1 (6.65–7.58)	7.14 (6.71–7.59)	7.09 (6.69–7.52)	6.99 (6.6–7.4)	0.449
Diagnosed hypertension	67.71 (66.6–68.81)	69.32 (68.33–70.28)	71.26 (70.38–72.13)	73.06 (72.25–73.85)	74.99 (74.25–75.72)	76.1 (75.4–76.78)	77.26 (76.6–77.91)	<0.001
**CONTROL AND TREATMENT** *								
**TOTAL**	**(n = 6611)**	**(n = 8440)**	**(n = 10163)**	**(n = 11837)**	**(n = 13435)**	**(n = 14918)**	**(n = 16116)**	
Blood pressure <130/80 mmHg	16.71 (15.91–17.51)	19.51 (18.64–20.37)	21.7 (20.78–22.61)	24.22 (23.26–25.19)	22.69 (21.75–23.62)	25.75 (24.75–26.68)	28.73 (27.68–29.78)	<0.001
Blood pressure <140/90 mmHg	53.03 (51.61–54.46)	56.36 (54.89–57.83)	59.15 (57.64–60.66)	61.64 (60.1–63.18)	59.16 (57.65–60.67)	62.60 (61.05–64.15)	65.79 (64.2–67.38)	<0.001
Antihypertensive drugs	97.7 (95.76–99.64)	97.43 (95.49–99.36)	97.32 (95.39–99.26)	97.11 (95.18–99.04)	97 (95.07–98.93)	96.85 (94.92–98.78)	96.69 (94.77–98.62)	<0.001
**FEMALES**	**(n = 3899)**	**(n = 4923)**	**(n = 5835)**	**(n = 6705)**	**(n = 7479)**	**(n = 8192)**	**(n = 8768)**	
Blood pressure <130/80 mmHg	16.69 (15.89–17.49)	18.7 (17.85–19.55)	21.16 (20.26–22.06)	23.34 (22.39–24.29)	22.77 (21.84–23.71)	25.11 (24.13–26.05)	28.18 (27.14–29.22)	<0.001
Blood pressure <140/90 mmHg	53.05 (51.62–54.48)	56.42 (54.95–57.9)	59.15 (57.64–60.66)	61.42 (59.89–62.96)	59.42 (57.91–60.93)	62.89 (61.33–64.44)	65.58 (63.99–67.17)	<0.001
Antihypertensive drugs	98.13 (96.19–100.07)	97.93 (95.99–99.87)	97.79 (95.85–99.73)	97.63 (95.69–99.56)	97.57 (95.63–99.5)	97.5 (95.56–99.43)	97.34 (95.41–99.28)	0.001
**MALES**	**(n = 2712)**	**(n = 3517)**	**(n = 4328)**	**(n = 5132)**	**(n = 5956)**	**(n = 6726)**	**(n = 7348)**	
Blood pressure <130/80 mmHg	16.74 (15.94–17.54)	20.7 (19.81–21.59)	22.45 (21.52–23.38)	25.44 (24.45–26.43)	22.57 (21.64–23.5)	26.56 (25.55–27.49)	29.43 (28.36–30.49)	<0.001
Blood pressure <140/90 mmHg	53.01 (51.58–54.44)	56.27 (54.8–57.74)	59.15 (57.64–60.65)	61.94 (60.4–63.48)	58.82 (57.32–60.32)	62.24 (60.7–63.79)	66.05 (64.46–67.64)	<0.001
Antihypertensive drugs	97.09 (95.16–99.02)	96.73 (94.8–98.66)	96.7 (94.77–98.62)	96.43 (94.51–98.36)	96.29 (94.37–98.21)	96.06 (94.14–97.98)	95.92 (94–97.84)	<0.001
**AGE <65 years**	**(n = 1970)**	**(n = 2478)**	**(n = 2824)**	**(n = 3172)**	**(n = 3516)**	**(n = 3749)**	**(n = 3858)**	
Blood pressure <130/80 mmHg	17.23 (16.42–18.05)	19.1 (18.25–19.96)	20.49 (19.6–21.38)	22.6 (21.67–23.53)	20.39 (19.5–21.27)	21.74 (20.83–22.63)	24.15 (23.19–25.12)	<0.001
Blood pressure <140/90 mmHg	56.04 (54.58–57.51)	58.19 (56.7–59.69)	59.62 (58.11–61.13)	62.06 (60.52–63.61)	59.19 (57.68–60.7)	61.56 (60.02–63.1)	64.21 (62.64–65.78)	<0.001
Antihypertensive drugs	97.11 (95.18–99.04)	96.85 (94.92–98.78)	96.53 (94.6–98.45)	96.03 (94.11–97.95)	95.96 (94.04–97.88)	95.39 (93.47–97.3)	95 (93.09–96.91)	<0.001
**AGE ≥65 years**	**(n = 4641)**	**(n = 5962)**	**(n = 7339)**	**(n = 8665)**	**(n = 9919)**	**(n = 11169)**	**(n = 12258)**	
Blood pressure <130/80 mmHg	16.5 (15.7–17.29)	19.66 (18.79–20.53)	22.13 (21.2–23.05)	25.11 (24.13–26.09)	23.43 (22.48–24.38)	27 (25.98–27.94)	30.06 (28.98–31.13)	<0.001
Blood pressure <140/90 mmHg	51.84 (50.43–53.25)	55.66 (54.2–57.12)	58.98 (57.48–60.49)	61.50 (59.96–63.04)	59.15 (57.64–60.66)	62.93 (61.37–64.48)	66.25 (64.65–67.84)	<0.001
Antihypertensive drugs	97.95 (96.01–99.89)	97.67 (95.73–99.61)	97.63 (95.69–99.57)	97.51 (95.57–99.44)	97.37 (95.43–99.3)	97.34 (95.41–99.27)	97.23 (95.29–99.16)	0.002

* In patients with diagnosed hypertension.

Analysis of lineal trends shows an upward statistically significant trend for prevalence of diagnosed HTN, and a negative trend for undiagnosed HTN with BP between 130/80 mmHg and 140/90 mmHg. No trend is observed for the prevalence of undiagnosed HTN with BP≥140/90 mmHg throughout the period 2003–2009.

**Figure 1:**
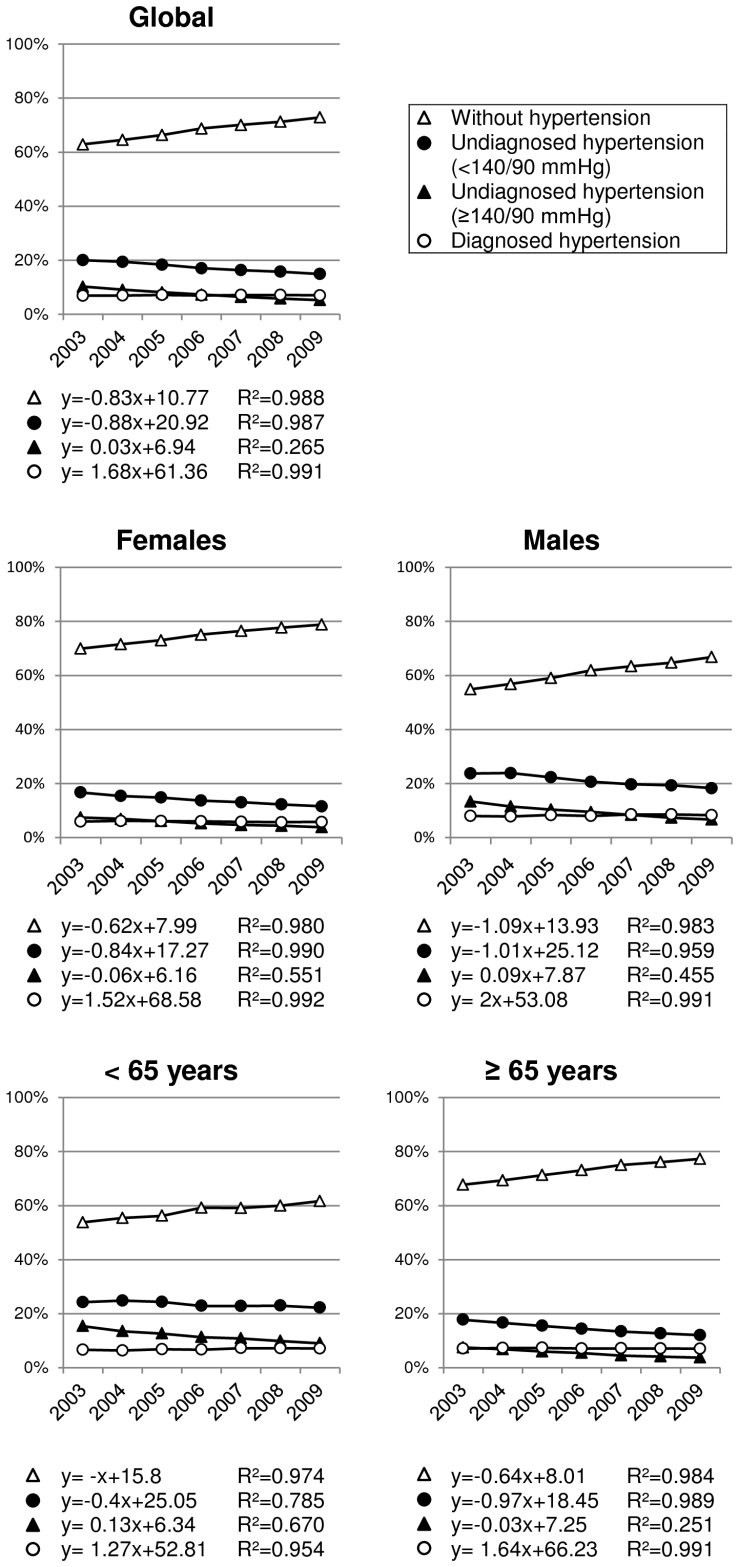
Time trend hypertension prevalence from 2003 to 2009.

99% of the variation observed in the prevalence can be explained by elapsed time, as shown by their coefficients of determination (R^2^). Figure 1 shows annual prevalence results with their regression line formulas.

The proportion of diagnosed HTN patients receiving pharmacological treatment remains around 97% throughout the entire study period (Table 2). The percentage of these patients who reached the BP objective showed a constant increase totalling 72.49% during the study period, reaching 28.79% in 2009.

Table 3 shows the control of BP for diagnosed HTN patients who received antihypertensive treatment and the type of drugs they were prescribed.

**Table 3:** Type of antihypertensive drugs and control among adults with diabetes and diagnosed hypertension receiving antihypertensive treatment between 2003 and 2009.

	2003	2004	2005	2006	2007	2008	2009	Trend test
	% (n = 6459)	% (n = 8223)	% (n = 9891)	% (n = 11495)	% (n = 13032)	% (n = 14448)	% (n = 15583)	p-value
Blood pressure control <140/90 mmHg	52.75 (51.33–54.17)	56.03 (54.56–57.49)	58.83 (57.33–60.33)	61.44 (59.9–62.98)	58.91 (57.4–60.41)	62.46 (60.91–64.01)	65.77 (64.18–67.36)	<0.001
Systolic blood pressure control <140 mmHg	54.03 (52.59–55.47)	57.24 (55.76–58.73)	59.89 (58.37–61.41)	62.33 (60.79–63.88)	59.91 (58.39–61.43)	63.33 (61.78–64.89)	66.57 (64.98–68.17)	<0.001
Diastolic blood pressure control <90 mmHg	91.45 (89.57–93.32)	92.34 (90.46–94.23)	93.23 (91.34–95.12)	93.34 (91.45–95.24)	93.12 (91.23–95.01)	94.24 (92.34–96.14)	94.66 (92.75–96.57)	<0.001
Blood pressure control <130/80 mmHg	16.69 (15.89–17.49)	19.36 (18.50–20.22)	21.63 (20.72–22.54)	24.10 (23.14–25.06)	22.58 (21.65–23.51)	25.74 (24.75–26.74)	28.79 (27.73–29.84)	<0.001
Systolic blood pressure control <130 mmHg	21.81 (20.90–22.73)	24.15 (23.19–25.11)	26.53 (25.52–27.54)	29.1 0(28.04–30.15)	26.94 (25.92–27.96)	30.00 (28.93–31.08)	33.02 (31.89–34.15)	<0.001
Diastolic blood pressure control <80 mmHg	50.23 (48.84–51.61)	56.03 (54.56–57.49)	58.13 (56.64–59.63)	60.27 (58.75–61.79)	59.64 (58.13–61.16)	63.07 (61.52–64.63)	65.80 (64.21–67.39)	<0.001
Diuretics	83.13 (81.35–84.92)	82.07 (80.29–83.84)	81.22 (79.46–82.99)	80.68 (78.92–82.44)	79.72 (77.97–81.47)	78.46 (76.72–80.19)	77.55 (75.82–79.27)	<0.001
Beta-blockers	16.33 (15.53–17.12)	17.58 (16.76–18.41)	19.33 (18.47–20.19)	20.74 (19.85–21.63)	22.26 (21.34–23.19)	23.28 (22.33–24.22)	24.06 (23.10–25.02)	<0.001
Calcium channel blockers	53.89 (52.45–55.33)	51.78 (50.37–53.20)	49.72 (48.34–51.10)	48.78 (47.41–50.15)	47.72 (46.36–49.07)	47.02 (45.68–48.37)	45.82 (44.49–47.15)	<0.001
Renin–angiotensin system drugs	91,92 (90,18–93,65)	91,52 (89,78–93,25)	91,56 (89,83–93,3)	91,28 (89,55–93,01)	91,26 (89,53–92,99)	91,03 (89,3–92,76)	90,9 (89,17–92,62)	0.008
− ACE inhibitors	78.44 (76.71–80.18)	78.51 (76.77–80.25)	78.36 (76.62–80.09)	77.86 (76.14–79.59)	77.83 (76.1–79.56)	77.71 (75.98–79.44)	77.35 (75.63–79.07)	0.019
− Angiotensin II receptor blockers	23.15 (22.20–24.09)	25.91 (24.92–26.91)	38.10 (36.89–39.31)	29.50 (28.44–30.57)	32.52 (31.4–33.64)	34.9 (33.74–36.05)	36.41 (35.23–37.59)	<0.001
Other antihypertensive drugs	17.34 (16.52–18.15)	16.48 (15.68–17.27)	15.61 (14.84–16.39)	14.96 (14.20–15.72)	14.17 (13.43–14.91)	13.86 (13.13–14.59)	13.43 (12.71–14.14)	<0.001
Two or more antihypertensive drugs	56.43 (54.96–57.90)	65.27 (63.68–66.85)	66.13 (64.54–67.73)	64.13 (62.56–65.70)	63.54 (61.98–65.1)	63.13 (61.58–64.69)	62.45 (60.91–64.00)	0.211
N° of antihypertensive drugs Mean (SD)	2.72 (1.12)	3.33 (1.63)	3.41 (1.69)	3.30 (1.65)	3.29 (1.67)	3.30 (1.70)	3.27 (1.70)	

The average number of antihypertensive drugs prescribed per patient rose from 2.72 in 2003 to 3.27 in 2009.

The groups of antihypertensive drugs most prescribed were renin-angiotensin system drugs followed by diuretics, calcium channel blockers and by beta-blockers.

During the study period prescriptions for angiotensin II receptor blockers were those which most increased, by 57.29%. The use of calcium channel blockers decreased by 14.97%, of diuretics by 6.72% and of ACE inhibitors by 1.39%.

The prevalence of HTN, awareness, treatment and control estimated with the cut-off point fixed in diagnostic criteria established by the European Guide to the Management of HTN in November 2009 are shown in the Supporting Information as Table S1.

## Discussion

The population included in the study has socio-demographic and clinical characteristics similar to other studies undertaken in PC in Spain during the same years [20]–[24].

### Prevalence

The results of this study show that a large majority of patients with type 2 diabetes treated in PC suffer HTN. These results are higher than those of other studies carried out in PC in Spain, which find prevalence between 80% and 84% [19], [25], and also show higher prevalence for women and for patients over 65 years old.

The upward trend in HTN prevalence has also been observed in diabetic patients of other countries [26], [27], as also is the case for the general population [3], [28]–[32].Under-diagnosis of HTN is a well-known phenomenon in the general population [31], [33], [34] and also in patients with diabetes [25], [27], [35]. The percentage of patients with HTN who were aware of their hypertension increased by 22.9% during the study period. This increase was greater for women and for patients over 65 in spite of starting from a more favourable situation, coinciding with previous studies [3], [31], [32].

Although there was a significant rising trend in the diagnosis of HTN, there is still a large margin for improvement since 23.13% of patients remained undiagnosed in 2009. This percentage is higher than the 20.6% found in the DIAPA study [25], also carried out with diabetic patients in Spain. This difference could be due, to the diagnostic threshold in that study being set at 130/85 mmHg, unlike ours which was set at 130/80, or, to both the doctors and the patients in the DIAPA study were volunteers, unlike the case of our study which included all patients and doctors in the PC Health Centres.

Under-diagnosis of HTN was found mainly in the group of patients with BP<140/90 mmHg, who represented over two thirds of undiagnosed patients. Furthermore, in this subgroup of patients the under-diagnosis diminished significantly between 2003 and 2009, while the proportion of patients with BP≥140/90 remained constant at values around 7%.

It is possible that some doctors may have been using the cut-off points for HTN diagnosis for the general population (≥140/90 mmHg) [12] for people with diabetes, and that over time, their awareness or level of agreement with the clinical guides’ recommendations increased and therefore their diagnoses may have improved. This would justify that the passage of time explains 99% of the variation found between 2003 and 2009.

### Control and Treatment

The control of BP in diabetic patients with diagnosed HTN, although still far from optimum, improved considerably reaching 28.73% in 2009. If we take as control criterion the objective of 140/90 mmHg, the control level in 2003 was 53.03%, a value slightly lower than the 58.6% found in the CONTROLPRES study [36], done with this cut-off point in the same year for the Spanish population attended in PC. The poor control of HTN for people with type 2 diabetes, due mainly to difficulty in reaching systolic BP objectives [20], [23]–[25], [27], [28], [32], [37], as well as the progressive improvement as time passes [20], [30], [32] are consistent with findings of other studies.

The proportion of HTN patients under treatment is very high, more than 96% of patients taking prescribed antihypertensive drugs, as also shown by other Spanish studies [23]–[25].

The results of the HOT [8] and UKPDS [9] studies show that to reach the BP objectives, the majority of patients require more than two antihypertensive drugs. In our study, 56.43% of patients under treatment were taking more than two antihypertensive drugs in 2003 and this percentage rose to 62.45% in 2009. These results are better than those found in other studies, where the proportion of patients taking more than two drugs was found to be between 13.3% and 26.8% [20], [23], [24]. This could be due to the fact that all antihypertensive drugs prescribed by family doctors were included in our study, although treatment may initially have been prescribed for another condition, such as renal protection, heart failure, stroke, arrhythmia, peripheral vascular disease or prostatic hyperplasia. Moreover, our analysis only includes patients with a diagnosis of HTN in their CCR. If we consider both diagnosed HTN patients as well as those who although suffering HTN were undiagnosed, the percentage falls to 40.34% in 2003 and to 47.15% in 2009.

As is the case in other studies in our field, the groups of antihypertensive drugs most prescribed were renin-angiotensin system drugs followed by diuretics [24], [25], [36]. Within the group of renin inhibitors a gradual rise in the use of angiotensin II receptor blockers and a slight fall in the use of ACE inhibitors were observed [29], [36]. An increase in the prescription of beta-blockers and a decrease in calcium channel blockers and diuretics is also recognisable [29], [36].

The improvement in BP control among diagnosed HTN patients was produced in spite of the percentage of patients under treatment and the average number of drugs used remaining almost unchanged. This could be due to the increase in numbers of patients taking more than three antihypertensive drugs, to the use of higher doses or to more effective combinations of drugs.

The discrepancy between the high percentage of patients under treatment and the poor control of BP may be attributed to different factors: on the part of patients, to the lack of adherence to pharmacological treatment and to changes in life-style; on the part of health professionals to the prescription of unsuitable or ineffective treatment, and lack of awareness or acceptance of the recommendations of the clinical practice guides. Several works have discovered that it is normal practice for doctors to begin anti-hypertensive treatment at BP values higher than those recommended in the clinical practice guides [38]–[40], that they are reluctant to intensify treatment in order to reach the desired BP [41], that they do not comply with recommendations when choosing the first-line drug [39]–[41] and, furthermore, tend to overvalue the effectiveness of the medical care which they offer [41].

On the other hand, it must be taken into account that diabetic patients are more sensitive to the vasoconstriction activity of angiotensin II, noradrenaline and salt. In addition, they are older [19], [23], more obese [23], suffer more complications and have stricter BP objectives than patients without diabetes.

### Strengths and Limitations

The Spanish National Health System offers coverage to over 95% of the population [42] and drugs prescribed are partially or wholly financed, chronic patients normally visiting Health Centres to receive prescriptions; for this reason we consider that the proportion of patients who may not have been included in our study to be low. Our study compiles available information on all the population attended in Health Centres, and by all health professionals, thus avoiding the possibility of bias associated with the participation of volunteers which occurs in other studies [23]–[25], [27], [29], [31], [32].

On the other hand, our work may have a classification bias similar to that reported in other studies [35], given that our definition of patients under treatment also included patients who were taking antihypertensive drugs for reasons other than HTN, as previously mentioned. In addition, a proportion of patients with undiagnosed HTN were having antihypertensive treatment, which could have resulted in an under-diagnosis of HTN.

Another possible limitation derives from the use of secondary information sources, the CCR. Electronic Health Records provide great potential for research, because of their ability to provide data for large populations. Even though the CCR can be used for research, it is important to note that the data were collected primarily for routine clinical rather than for researching purposes. In order to prevent compromising the results of studies, data quality and reliability were assessed previously by researchers by validating the diagnosis of HTN and diabetes coded in the CCR [19]. Nevertheless, the variables used seem robust, given that both the diagnoses as well as the BP measurements are frequently employed by family doctors and the quality of medical care depends, in part, on their correct recording.

The prevalence of HTN in the Spanish type 2 diabetes population found in our study is the highest of any published up to now, only 5.2% of diabetic patients showing BP values below 130/80 mmHg.

## Conclusions

Significant improvements have taken place in the diagnosis and the control level of HTN for people with type 2 diabetes, although these continue to be far from optimum, 23.13% of HTN patients remaining undiagnosed and only 28.79% of patients under treatment showing BP below 130/80 mmHg and 65.77% BP below 140/90 mmHg.

## Supporting Information

Table S1 **Prevalence, awareness, treatment and control of hypertension among adults with diabetes by sex and age groups between 2003 and 2009 with the diagnostic criteria established the European Guide to the Management of HTN in November 2009.**
(DOCX)
