# Supplementary material for: Trends in Hypertension Prevalence, Awareness, Treatment and Control in an Adult Type 2 Diabetes Spanish Population between 2003 and 2009
Source: PLoS One. 2014 Jan 27;9(1):e86713. doi: 10.1371/journal.pone.0086713 (PMC3903577; doi:10.1371/journal.pone.0086713)
Supplement: Table S1 — Prevalence, awareness, treatment and control of hypertension among adults with diabetes by sex and age groups between 2003 and 2009 with the diagnostic criteria established the European Guide to the Management of HTN in November 2009. (DOCX) [file pone.0086713.s001.docx]

|  |  | **2003**  **% (CI 95%)** | **2004**  **% (CI 95%)** | **2005**  **% (CI 95%)** | **2006**  **% (CI 95%)** | **2007**  **% (CI 95%)** | **2008**  **% (CI 95%)** | **2009**  **% (CI 95%)** | **Trend Test**  **P-Value** |
| --- | --- | --- | --- | --- | --- | --- | --- | --- | --- |
| **PREVALENCE AND AWARENESS** | **TOTAL** | **(n=10517)** | **(n=13075)** | **(n=15323)** | **(n=17221)** | **(n=19177)** | **(n=20934)** | **(n=22123)** |  |
|  | Without hypertension | 27,21 (26,19-28,24) | 26,77 (25,75-27,78) | 25,93 (24,93-26,93) | 24,38 (23,42-25,35) | 22,83 (21,89-23,77) | 22,66 (21,73-23,59) | 21,45 (20,54-22,36) | <0.001 |
|  | Undiagnosed hypertension (≥140/85 mmHg) | 9,93 (9,31-10,54) | 8,68 (8,1-9,26) | 7,75 (7,2-8,29) | 6,88 (6,37-7,4) | 7,11 (6,59-7,64) | 6,08 (5,59-6,56) | 5,7 (5,24-6,17) | <0.001 |
|  | Diagnosed hypertension | 62,86 (61,31-64,41) | 64,55 (62,98-66,13) | 66,33 (64,73-67,92) | 68,74 (67,11-70,36) | 70,06 (68,42-71,7) | 71,26 (69,61-72,92) | 72,85 (71,17-74,52) | <0.001 |
|  | **FEMALES** | **(n=5576)** | **(n=6883)** | **(n=7993)** | **(n=8929)** | **(n=9782)** | **(n=10544)** | **(n=11126)** |  |
|  | Without hypertension | 21,52 (20,61-22,43) | 21,27 (20,37-22,17) | 20,81 (19,91-21,7) | 19,73 (18,86-20,61) | 18,27 (17,43-19,11) | 17,9 (17,07-18,73) | 16,6 (15,8-17,4) | <0.001 |
|  | Undiagnosed hypertension (≥140/85 mmHg) | 8,55 (7,98-9,13) | 7,21 (6,68-7,73) | 6,19 (5,71-6,68) | 5,59 (5,13-6,05) | 5,27 (4,82-5,73) | 4,41 (4-4,82) | 4,59 (4,17-5,01) | <0.001 |
|  | Diagnosed hypertension | 69.92 (68.29-71.56) | 71.52 (69.87-73.18) | 73.00 (71.33-74.68) | 75.09 (73.39-76.79) | 76.46 (74.74-78.17) | 77.69 (75.97-79.41) | 78.81 (77.07-80.55) | <0.001 |
|  | **MALES** | **(n=4941)** | **(n=6192)** | **(n=7330)** | **(n=8292)** | **(n=9395)** | **(n=10390)** | **(n=10997)** |  |
|  | Without hypertension | 33,64 (32,5-34,77) | 32,88 (31,76-34) | 31,51 (30,41-32,61) | 29,87 (28,8-30,94) | 27,58 (26,55-28,61) | 27,5 (26,47-28,53) | 26,35 (25,35-27,36) | <0.001 |
|  | Undiagnosed hypertension (≥140/85 mmHg) | 11,48 (10,81-12,14) | 10,32 (9,69-10,95) | 9,44 (8,84-10,04) | 8,24 (7,67-8,8) | 9,03 (8,44-9,61) | 7,77 (7,22-8,31) | 6,83 (6,32-7,34) | <0.001 |
|  | Diagnosed hypertension | 54.89 (53.44-56.34) | 56.80 (55.32-58.28) | 59.05 (57.54-60.55) | 61.89 (60.35-63.43) | 63.40 (74.9-78.02) | 64.74(63.16-66.3) | 66.82 (65.22-68.42) | <0.001 |
|  | **AGE <65 years** | **(n=3663)** | **(n=4474)** | **(n=5024)** | **(n=5361)** | **(n=5950)** | **(n=6257)** | **(n=6257)** |  |
|  | Without hypertension | 34,75 (33,6-35,91) | 33,89 (32,75-35,03) | 33,18 (32,05-34,31) | 30,68 (29,6-31,77) | 29,02 (27,97-30,08) | 28,63 (27,58-29,68) | 27,07 (26,05-28,09) | <0.001 |
|  | Undiagnosed hypertension (≥140/85 mmHg) | 11,47 (10,8-12,13) | 10,54 (9,9-11,18) | 9,15 (8,56-9,74) | 8,33 (7,76-8,89) | 9,07 (8,48-9,66) | 8,15 (7,59-8,71) | 7,55 (7,01-8,09) | <0.001 |
|  | Diagnosed hypertension | 53.78 (52.16-55.39) | 55.39 (53.93-56.84) | 56.21 (54.83-57.58) | 59.17 (57.85-60.48) | 59.09 (60.34-57.84) | 59.92 (61.12-58.7) | 61.66 (62.86-60.45) | <0.001 |
|  | **AGE ≥65 years** | **(n=6854)** | **(n=8601)** | **(n=10299)** | **(n=11860)** | **(n=13227)** | **(n=14677)** | **(n=15866)** |  |
|  | Without hypertension | 23,18 (22,24-24,13) | 22,64 (21,7-23,57) | 21,43 (20,52-22,34) | 20,26 (19,38-21,15) | 18,42 (17,57-19,26) | 18,09 (17,26-18,93) | 16,97 (16,16-17,77) | <0.001 |
|  | Undiagnosed hypertension (≥140/85 mmHg) | 9,1 (8,51-9,7) | 7,6 (7,06-8,14) | 6,87 (6,36-7,39) | 5,93 (5,46-6,41) | 5,72 (5,25-6,18) | 4,49 (4,07-4,9) | 4,23 (3,83-4,63) | <0.001 |
|  | Diagnosed hypertension | 67.71 (66.6-68.81) | 69.32 (68.33-70.28) | 71.26 (70.38-72.13) | 73.06 (72.25-73.85) | 74.99 (74.25-75.72) | 76.1 (75.4-76.78) | 77.26 (76.6-77.91) | <0.001 |
| **CONTROL AND TREATMENT*** | **TOTAL** | **(n=6611)** | **(n=8440)** | **(n=10163)** | **(n=11837)** | **(n=13435)** | **(n=14918)** | **(n=16116)** |  |
|  | Blood pressure <140/85 mmHg | 48.05 (46.69-49.41) | 57.62 (56.13-59.11) | 55.32 (53.86-56.77) | 58.08 (56.58-59.57) | 55.7 (54.24-57.16) | 59.36 (57.85-60.87) | 62.7 (61.15-64.26) | <0.001 |
|  | **FEMALES** | **(n=3899)** | **(n=4923)** | **(n=5835)** | **(n=6705)** | **(n=7479)** | **(n=8192)** | **(n=8768)** |  |
|  | Blood pressure <140/85 mmHg | 48.71 (47.34-50.08) | 52.43 (51.01-53.85) | 55.82 (54.36-57.29) | 58.42 (56.92-59.92) | 56.32 (54.85-57.79) | 64.92 (63.34-66.49) | 62.74 (61.18-64.29) | <0.001 |
|  | **MALES** | **(n=2712)** | **(n=3517)** | **(n=4328)** | **(n=5132)** | **(n=5956)** | **(n=6726)** | **(n=7348)** |  |
|  | Blood pressure <140/85 mmHg | 47.05 (45.71-48.4) | 52.13 (50.72-53.55) | 54.61 (53.16-56.06) | 57.61 (56.12-59.1) | 54.87 (53.42-56.32) | 66.04 (64.45-67.64) | 62.66 (61.11-64.22) | <0.001 |
|  | **AGE <65 years** | **(n=1970)** | **(n=2478)** | **(n=2824)** | **(n=3172)** | **(n=3516)** | **(n=3749)** | **(n=3858)** |  |
|  | Blood pressure <140/85 mmHg | 46.46 (45.13-47.8) | 50.3 (48.91-51.69) | 51.38 (49.98-52.79) | 54.3 (52.85-55.74) | 51.6 (50.2-53.01) | 64.35 (62.77-65.92) | 56.7 (55.22-58.18) | <0.001 |
|  | **AGE ≥65 years** | **(n=4641)** | **(n=5962)** | **(n=7339)** | **(n=8665)** | **(n=9919)** | **(n=11169)** | **(n=12258)** |  |
|  | Blood pressure <140/85 mmHg | 48.68 (47.31-50.05) | 53.08 (51.66-54.51) | 56.71 (55.24-58.19) | 59.32 (57.81-60.83) | 57.02 (55.54-58.5) | 65.96 (64.36-67.55) | 64.44 (62.87-66.01) | <0.001 |

*In patients with diagnosed hypertension.

Table S1. Prevalence. awareness. treatment and control of hypertension among adults with diabetes by sex and age groups between 2003 and 2009 with the diagnostic criteria established the European Guide to the Management of HTN in November 2009.
